# 15 years of microbial biotechnology: the time has come to think big—and act soon

**DOI:** 10.1111/1751-7915.13993

**Published:** 2021-12-21

**Authors:** Víctor de Lorenzo

**Affiliations:** ^1^ Centro Nacional de Biotecnología CSIC Madrid Spain

## Abstract

Our epoch is largely characterized by the growing realization and concern about the reality of climate change and environmental deterioration, the surge of global pandemics, the unacceptable inequalities between developed and underdeveloped countries and their unavoidable translation into messy immigration, overpopulation and food crises. While all of these issues have a fundamentally political core, they are not altogether removed from the fact that Earth is primarily a microbial planet and microorganisms are the key agents that make the biosphere (including ourselves) function as it does. It thus makes sense that we bring the microbial world—that is the environmental microbiome—to the necessary multi‐tiered conversation (hopefully followed by action) on how to avoid future threats and how to make our globe a habitable common house. Beyond discussion on governance, such a dialogue has technical and scientific aspects that only frontline microbial biotechnology can help to tackle. Fortunately, the field has witnessed the onset of new conceptual and material tools that were missing when the journal started.

## Graphical abstract

Microbial Biotechnology enters a new period framed by the awareness of climate change and its consequences. Fortification of the environmental microbiome with a suite of agents with enhanced bioremediation activities (from monoclonal strains to self‐propagating DNA) will be paramount for reverting emissions to pre‐industrial levels.

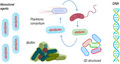

## A decade and a half of easy DNA sequencing—and its unexpected consequences

The one development that has revolutionized biological research of the past 15 years has been the growing ease and affordability of DNA sequencing (Shendure *et al*., 2017). This has been accompanied by a similar ease of access to complete transcriptomes, proteomes and a ramping number of other *omes* in all sorts of biological samples (Karahalil, 2016). The resulting avalanche of data has in turn created a need to process and distil them into useful information. Since handling huge volumes of data is clearly beyond capacities of individuals and even of traditional Bioinformatics and Systems Biology, it comes as little surprise that Artificial Intelligence (AI) and Machine Learning (ML)—and other purely computational methods—are in many cases replacing traditional hypothesis‐driven approaches to tackle biological questions (Gilpin *et al*., 2020). The recently developed AlphaFold platforms (AI systems developed by DeepMind that predicts a protein’s 3D structure straight from its amino acid sequence; Senior *et al*., 2020) clearly illustrate this point, as the accuracy they delivers is competitive with actual experiments. Alas, unlike DNA, high‐throughput determination of protein sequences directly is still a fastidious endeavour, which typically relies on sophisticated mass spectrometry techniques and very expensive equipment. But this may also change soon as ongoing research on applying nanopore technology to the proteome may enable reading primary protein sequences at the single‐molecule level (Brinkerhoff *et al*., 2021; Ying, 2021). In a different direction, computational methods are also helping to obtain better automated annotations of genomic data (Lai *et al*., 2018), to improve gene ontologies and to comprehend and model higher level functions (e.g. metabolic and regulatory networks) of single strains and complex communities (Thompson *et al*., 2019; Ghannam and Techtmann, 2021). One intriguing outcome of applying AI approaches to these scenarios is the detection of research issues that had not been formulated before and the answering of questions that had not been knowingly raised. The ever‐growing computational power available to researchers supports elaboration of both descriptive and predictive models that capture the key components of live entities from single cells to complete ecosystems. Metabolic models of a whole range of biological systems have been particularly useful (Gudmundsson and Nogales, 2021), and they hold a promise for guiding implementation of a suite of biotechnological interventions of the sort advocated below (García‐Jiménez *et al*., 2021). Finally, of considerable interest to microbial biotechnology is the recent development of ML platforms to support roadmaps for engineering heterologous gene expression workflows (Reis and Salis, 2020; Nikolados *et al*., 2021). While these approaches currently fail to provide mechanistic insights, they may solve practical problems that are not comprehensible with the principles known at the present time. It is thus likely that the whole field will growingly depend on computation and data in addition to the reliance on Molecular Biology knowledge that has dominated the biotech arena for decades.

## Genetic engineering: from analogy to authentic methodology

The ease of DNA sequencing and all the downstream consequences just discussed has grown in parallel to equally uncomplicated access to chemical synthesis of nucleic acids. This has enabled and accelerated straightforward construction of genetic devices—all the way to complete genomes (Venetz *et al*., 2019)—way more easily than using lengthy cloning of DNA and manual assembly methods. Production of customized ever‐longer DNA segments has been one of the main technical drivers of contemporary Synthetic Biology, a framework that looks at live systems through the eyes and relational logic of electric, mechanical and process engineering. However, Synthetic Biology is not just an analogy (as in the case of Genetic Engineering) but it is a veritable instrument to both understand biological phenomena and reshape activities with given specifications at a user’s will (Heinemann and Panke, 2006). The ambition to build and programme live systems with the straightforwardness that an engineer designs and manufactures human‐made artefacts clashes with two realities of biological systems: the context dependence of performance and the inevitable evolution and mutability that are inherent to any DNA‐encoded trait (Kwok, 2010). Given that such phenomena are determined by a very large number of factors which are often beyond our present technical and theoretical grasp, the current trend is again turning to massive data generation, AI and ML to guide the engineering of live entities. One means to this end involves the set‐up of the so‐called biofoundries, that is large‐scale technological platforms for DNA synthesis and experimentation that enable generation and assessment of huge numbers of genetic constructs (Hillson *et al*., 2019; Holowko *et al*., 2021). These biofoundries may thus shorten for Synthetic Biology the typical design‐build‐test‐learn (DBTL) cycle that is standard in engineering. While this approach may not provide answers to basic biological questions, it offers useful roadmaps to specific biotechnological projects, and it is thus likely to remain as a favourite line of attack to otherwise intractable bio‐design challenges. A side benefit of bringing *bona fide* engineering to biotechnology is the growing interest in technical and semantic standards for overcoming the soft, mostly qualitative and highly metaphorical narrative of biological phenomena. In this sense, the last few years have witnessed many propositions for not only standardizing genetic tools and DNA assembly methods but also advocating a rigorous metrology of archetypal biological activities and adoption of machine‐readable description of genetic constructs (Beal *et al*., 2020). All these developments break with the idealization of Biology as something different from the rest of the hard sciences and place Biotechnology in the orbit of authentic Engineering rather than remaining a spinoff of Molecular Biology (Porcar *et al*., 2015).

In sum, it seems that a number of conceptual and material assets like the ones just mentioned (and many others) are now ripe for bringing Microbial Biotechnology to a new level of efficacy in order to solve problems and offer new products and services. But which of these could be the most important?

## Metabolic engineering: not just the pathway

While the use of microorganisms for production of valuable chemicals predates the recombinant DNA era, rational genetic assembly of metabolic routes, whether for catabolism of given compounds or synthesis of valuable molecules, has been one of the most successful branches of contemporary Biotechnology (Na *et al*., 2010; Choi *et al*., 2019). Design of a pathway that produces artemisinic acid in yeast (Ro *et al*., 2006) is often considered a turning point in the chronicle of metabolic engineering. This historical importance is not only because of the many genes and genetic modifications involved, but also for the adoption of Systems and Synthetic Biology approaches and optimization strategies much closer to the DBTL tenets of engineering than the somewhat naïve trial‐and‐error methods of earlier stages (Paddon and Keasling, 2014). Since then, production of a plethora of added‐value compounds has been realized based on metabolic models, advanced genetic tools (many of them built on CRISPR‐centred parts), synthetic DNA (Smanski *et al*., 2014) and adaptive laboratory evolution (ALE; Portnoy *et al*., 2011). A large number of bioinformatic and computational platforms are easily accessible for automated construction of pathways in silico for synthesis or degradation of target molecules. These platforms offer the user a range of genes that can be collected and combined to that end (Woodruff *et al*., 2017; Lin *et al*., 2019; Hafner *et al*., 2020). In a subsequent screw turn, the *control* of such synthetic pathways can also be computationally designed *á la carte*, as circuits of Boolean logic gates implemented with transcriptional factors and regulatory DNA sequences (Nielsen *et al*., 2016). There have been significant parallel efforts as well to optimize production hosts by moving to microorganisms other than *E. coli* as the recipients of the recombinant pathways (Adams, 2016). This in turn has triggered a growing attention to developing standardized strains as genetic and physiological chassis for optimizing specific transformations and other bioproduction processes (de Lorenzo *et al*., 2021). Interestingly, there has been a crescent realization that streamlining of a whole‐cell catalyst is not just about genetically augmenting expression of the genes or pathways at stake. The physical shape of the active biological agents, their tolerance to stress and their genetic stability make a considerable difference in their performance. It is thus not surprising that several present‐day efforts in metabolic engineering are directed not so much to pathway optimization as to physical fortification of the chassis, extending their active lifespan and performance in non‐conventional settings (Volke and Nikel, 2018). Such endeavours are supported by ongoing attempts to comprehend bacterial cells not as mere recipients of genes/DNA and enzymes, but as highly structured physicochemical nanomachines that—similarly to factories—adopt an inner 3D architecture of channels, pumps and valves that optimize performance (Llopis *et al*., 2010). Contemporary biology has been so gene‐centred thus far that we often neglect that cells are run by physical principles and a relational logic reminiscent of chemical factories. A better understanding of this can result not just in new molecules but also in smart biological goods in which the product of the biotechnological process is the biomass itself, for example bacterial leather, smart bio‐textiles and a range of functional biomaterials (Gilbert and Ellis, 2018). In contrast with such rapid advances in the blueprint of whole‐cell catalysts, there is still much to do for improving bioreactor design, the basic layout of which (a vessel with a sterile liquid culture inoculated with a single strain) has remained virtually identical for too long.

## The way back from isolates to microbiomes

If the birth of modern Microbiology is often associated with Koch’s methods to isolate and grow bacteria as clonal strains with individual properties, the last decade (again owing to the ease of DNA sequencing of metagenomes and other *omics*) has witnessed a vast shift in exactly the opposite direction: a realization of microbiomes (including viromes; Paez‐Espino *et al*., 2016) and microbial consortia as the protagonists of everything that matters in the biological world – from individual humans to large ecosystems (Małyska *et al*., 2019; Zhu and Penuelas, 2020; Nayfach *et al*., 2021). This notion has been extensively substantiated and well noted by the biotechnological community, which has become increasingly aware of the biomedical and therapeutic promise—and sure profits—of microbiome studies. To this end, a large number of both *in silico* and wet‐lab technologies have been developed for extracting activities and interesting biomolecules from naturally occurring bacterial consortia and microbiomes of all sorts (van der Helm *et al*., 2018). On the other hand, Synthetic Biology enables design of probiotic strains and formulations of therapeutic value for medical practice and animal farming (Foo *et al*., 2017; McCarty and Ledesma‐Amaro, 2019). Note that such a rational design does not necessarily mean the use of recombinant DNA technology: An emerging branch of contemporary biotechnology is currently developing under the banner of *Genetic Engineering‐free Synthetic Biology* (Konstantinidis *et al*., 2021). This field advocates systems‐guided assembly of specific microbial partnerships and evolutionary adaptation towards given activities as an alternative roadmap to generate novel properties. This approach, largely inspired on recent work on the super‐stable microbial community of kefir (Blasche *et al*., 2021), is likely to yield excellent dividends in terms of both efficacy and public acceptance.

Engineered microbiomes also will find a fertile field of application in the agricultural sectors, where growth‐promoting and crop‐protecting live biologicals may outcompete genetic engineering of plants as a way to avoid the use of chemical fertilizers, production‐enhancers and pesticides (Ke *et al*., 2021). Interestingly, these developments may also converge with current efforts to design plants able to directly fix N_2_ from air. This longstanding dream of modern biotechnology (Vicente and Dean, 2017) looks now much more at hand owing to recent progress in effective expression of nitrogenase in eukaryotic cells (Burén *et al*., 2019; Eseverri *et al*., 2020). The agricultural revolution that such a technology could bring about may be comparable to that of the Haber‐Bosch reaction for chemical synthesis of ammonia. Lastly, the fact that major chemical processes in nature are run not by single species but by microbial consortia has inspired (and will continue to do so) the design of multi‐strain catalysts in which a complex process is split in separate tasks fulfilled by individual members of the partnership (Bassalo *et al*., 2016; McCarty and Ledesma‐Amaro, 2019). In fact, such multi‐strain catalysts endowed with a division of labour might ultimately do better than single agents: by manipulating the composition one can manage the stoichiometry and the activities of the biological materials through a much wider parameter space.

## New problems: new solutions?

While all the advances and prospects discussed above are veritable game changers, they occur in a context of global problems that were less evident when the journal was founded. At least three such threats are distressing our societies on a planetary, unprecedented level. This article is written at the time when the COVID‐19 pandemic still delivers successive waves of infections, which are only partially contained in wealthier countries through massive vaccination campaigns. While this still unfinished episode brought about by SARS CoV‐2 has exposed our defencelessness towards new viral or bacterial pathogens, it has also made evident the power of modern biotechnology to develop and put in place in very little time a suite of potential solutions. In particular, the efficacy and ease of production of RNA‐based vaccines was unthinkable just a few years earlier (Callaway, 2020). It is likely that the awareness of our vulnerability towards emerging pathogens elicited by COVID‐19 will not only increase interest in vaccines but also will ease regulatory frameworks and public concerns about engineered therapeutics. Furthermore, the dearth of drugs to combat SARS CoV‐2 has in turn placed a new and strong focus on the equally manifest shortage of new antibiotics to check the ramping growth of antimicrobial resistances (Aslam *et al*., 2018). In fact, very few authentically novel antimicrobials have made it to clinical practice in recent years. Ongoing, mostly academic efforts to cope with the problem involve surveying of peptide antibiotics (sometimes found in the most unexpected places; Torres *et al*., 2021), new functional tests for molecules with antimicrobial activity (Wrighton, 2018) or reliance on phages (Gordillo Altamirano and Barr, 2019). Other propositions for dealing with new pathogens and antibiotic resistances involve surveillance strains in the microbiome that conjugatively deliver plasmids able to activate host‐killing circuits when a virulence signal or a resistance gene is detected in a recipient bacterium (López‐Igual *et al*., 2019; Wongpayak *et al*., 2021). Whether such smart strategies will make it to actual patients remains an open question, but innovative ideas of this sort are badly needed and will surely emerge with more frequency in the foreseeable future.

## Towards large‐scale bioremediation

As indicated at the beginning of this Editorial, the single biggest problem that we encounter and will keep on facing as human society is climate change. It is noteworthy that frontline research on environmental biotechnology 15 years ago mostly dealt with site‐specific contamination, the chemicals at stake and the pathways/microorganisms able to deal with them. This is still a considerable task and will continue to be for years to come. Yet, the new question that has emerged is whether we can also capitalize on the environmental microbiome for the sake of controlling and ultimately reverting the global impact of emissions and pollution of anthropogenic origin on the functioning of the Earth’s biosphere. The current situation is characterized by a worrying increase of greenhouse gases, the massive pollution of aquatic ecosystems with plastics and the ramping expansion of drylands and scorched soil. These three go along with other extensive difficulties, for example the spread of micropollutants through water systems, the mismanagement of N and P, and the accumulation of lignocellulosic residues (de Lorenzo, 2017). There is good evidence that some such occurrences are pushing ecosystems well beyond a tipping point (Vidiella *et al*., 2020; Berdugo *et al*., 2021), which makes purely mitigation measures (e.g. reduction of emissions) already useless. In turn, this opens a challenge and an opportunity for frontline microbial biotechnology to explore the thus far uncharted territory of upscaling bioremediation interventions to an unprecedented dimension (de Lorenzo *et al*., 2016). The theoretical framework of this endeavour is related to the longstanding concept of Terraforming: can we bring life to a geochemical scenario that has thus far not supported biological systems? By the same token, could it be feasible to restore the functioning of ecosystems that have been destroyed by anthropogenic emissions (Conde‐Pueyo *et al*., 2020)? A number of models suggest that this could be the case, provided that self‐propagating biological agents with specified properties could be engineered and propagated at large scale (Solé *et al*., 2015). One such property is CO_2_ fixation, which is currently the subject of considerable research both in terms of enhancing naturally occurring C‐capture processes, for example photosynthesis, and designing entirely new routes to the same end (Claassens, 2017; Schada von Borzyskowski *et al*., 2018; Liang *et al*., 2020). A different type of activity towards the same concerns is biodegradation of synthetic plastic materials, a turning point in which was the discovery and molecular cloning of the PETases, an esterase class of enzymes that hydrolyze polyethylene terephthalate (PET) plastic to easier‐to‐degrade intermediates (Maity *et al*., 2021). PET hydrolysis seems to be widespread through the entire global microbiome (Zrimec *et al*., 2021) and perhaps degradation of other polymers as well (Solé *et al*., 2017). This opens prospects for dealing with the phenomenally large problem of plastic pollution.

Unfortunately, whether CO_2_ capture, plastic degradation, catabolism of micropollutants or other serious planet‐wide contamination issues, solutions are not found just by having the activity of interest or pathway working in the laboratory. The real challenge is to deliver such remedies at a very large scale (de Lorenzo *et al*., 2016). But how can this be brought about? From an implementation point of view, this question has three somewhat separate technical challenges: (i) artificially improving reactions of interests (or creating altogether new ones), (ii) effective expression of such engineered pathways in suitable hosts or consortia thereof and (iii) large‐scale propagation of the improved agents and activities through the environmental microbiome. Fortunately, these issues are tractable with the wealth of conceptual and material tools of contemporary Synthetic Biology. Obviously, they also touch upon questions on biosafety, public perception, governance etc. This is a first‐time technical challenge that may require considerable creativity, where effective performance will have to go hand in hand with safety measures to ensure that the benefits in a time for urgent action far exceed the potential risks.

## Cui prodest?

Since the birth of modern biotechnology, market forces have largely shaped the research priorities in the field. Many of the past and expected developments discussed above enjoy a clear roadmap to commercialization of bio‐based products and services. This is most clearly exemplified in the field of new therapies: novel treatments or new drugs for degenerative diseases have a high added value as well as an instant market demand with prospects of considerable profits for the particular companies which produce them. In these cases, large investments in R&D are fully justified from a market perspective, although the benefits are limited to those who can pay for the products. In contrast, the end beneficiaries of other branches of Biotechnology, in particular environmental biotech, are not individuals but large societal groups, which may not afford *directly* the cost of what they receive. Despite the urgent necessity of large‐scale CO_2_ removal from the atmosphere, the reality is that the actual economic incentives to develop technologies to that end do not stem directly from an *existing* market demand, but only from legislation that *creates* a market demand. Large‐scale bioremediation technologies are thus bound to be pushed by the public sector—or else they will not happen. The ongoing narrative on circular economy and valorisation of emissions and waste (instead of its mere destruction) may turn useless as the necessary financing to address global problems may not yield short‐term profits to investors. How these economic aspects of the research and actions necessary to tackle climate change will be handled remain a most critical issue for the future of our planet. Large‐scale environmental issues, in particular those related to emissions, are scientifically fascinating and undeniably critical for the larger World’s population. But the investment the field receives is a fraction of what other branches of Biotechnology (e.g. human‐health oriented) enjoy. But how to move priorities from being exclusively market‐dominated and profit‐driven to include also the benefit of the general public in connection to the climate crisis? Alas, not every waste can be converted into value to the levels required to have an impact on the balance of global emissions. Therefore we need to assume that advanced technologies to improve global environmental quality may be economically deficitary in the short run. In order to accept this reality, it is essential to increase awareness of public and business sectors that the impact of climate change will depend heavily on responses of microorganisms, which are essential for achieving an environmentally sustainable future: they are in fact our main if not the only ally to manage the current impasse (Cavicchioli *et al*., 2019). For this, an improvement of the microbiological literacy of the general population is badly needed (Timmis *et al*., 2019). The generalization of the Internet, the impact of social media in political decision‐making and the influence of NGOs in our times have set up new channels of instant and direct interplay both among scientists (including of course microbial biotechnologists) and between researchers and the general public. Such a connectivity has the potential to create new links and complicities among environmental stakeholders that have been traditionally way apart. Modern recombinant DNA‐based biotechnology has often alienated large societal sectors because of the emphasis on profit, disregard of the risks and advantages only to the few (Goven and Pavone, 2015). In contrast, efforts to curb climate change and many of its downstream consequences with biological tools can also show that another Biotechnology is not only possible but also desirable and necessary.

## Conflict of interest

Author declares no conflict of interest.
